# Activity of the Lactate Dehydrogenase Inhibitor Oxamic Acid against the Fermentative Bacterium *Streptococcus mitis/oralis*: Bactericidal Effects and Prevention of Daptomycin Resistance In Vitro and in an Ex Vivo Model

**DOI:** 10.3390/antibiotics11101409

**Published:** 2022-10-13

**Authors:** Razieh Kebriaei, Arnold S. Bayer, Christian K. Lapitan, Michael J. Rybak, Greg A. Somerville, Nagendra N. Mishra

**Affiliations:** 1 Anti-Infective Research Laboratory, Eugene Applebaum College of Pharmacy and Health Sciences, Detroit, MI 48202, USA; 2 Division of Infectious Diseases, The Lundquist Institute at Harbor-UCLA Medical Center, Torrance, CA 90502, USA; 3 The David Geffen School of Medicine, University of California, Los Angeles (UCLA), Los Angeles, CA 90095, USA; 4 School of Medicine, Wayne State University, Detroit, MI 48202, USA; 5 School of Veterinary Medicine and Biomedical Sciences, University of Nebraska-Lincoln, Lincoln, NE 68588-0905, USA

**Keywords:** oxamic acid, lactate dehydrogenase, daptomycin resistance

## Abstract

*Streptococcus mitis/oralis* is a fermentative bacterium that relies on lactate dehydrogenase to balance its redox poise and keep glycolysis active. Metabolomic analysis of an in vitro–derived daptomycin-resistant (DAP-R) *S. mitis/oralis* strain (351-D10) revealed differences in glucose catabolism relative to its DAP-susceptible (DAP-S) parental strain, 351. Metabolic changes associated with the transition to this DAP-R phenotype suggested that inhibiting glycolysis could alter DAP susceptibility. In addition, the strong reliance of *S. mitis/oralis* on glycolysis for energy and biosynthetic intermediates suggested that inhibiting glycolysis would adversely affect growth and biomass accumulation. To test these hypotheses, we used the lactate dehydrogenase inhibitor oxamic acid (OXA) to assess its efficacy against DAP-S *S. mitis/oralis* strain 351 during DAP exposures in vitro and ex vivo. As expected, OXA was growth inhibitory to *S. mitis/oralis* in a dose-dependent manner in vitro; however, it did not alter in vitro DAP susceptibility profiles. In contrast, OXA did prevent the emergence of DAP-R in an ex vivo model of simulated endocardial vegetations. These data suggest that metabolic inhibitors directed against this fermentative bacterium with limited metabolic capabilities could enhance killing and potentially forestall the emergence of DAP resistance.

## 1. Introduction

The viridans group streptococci (VGSs) are a heterogeneous group of pathogens that reside predominantly in the oropharynx and include the *Streptococcus mitis/oralis* subgroup. This latter subgroup is the most common etiology of infective endocarditis (IE) amongst the VGS, as well as the principle cause of the “toxic Strep syndrome” in neutropenic cancer patients [1,2,3,4,5,6]. *S. mitis/oralis* infections have become more difficult for physicians to treat due to the emergence of penicillin and cephalosporin resistance, as well as intrinsic vancomycin tolerance [7]. In the process of acquiring such antibiotic resistances, bacterial metabolism must adapt to facilitate these resistance mechanism(s) [8,9,10,11].

The above facts led us to examine the metabolic changes associated with the emergence of DAP-R in *S. mitis/oralis* [9]. We observed perturbations in glycolysis, the pentose phosphate pathway, and some amino acid biosynthetic pathways. Changes in glycolysis are important because VGSs preferentially ferment carbohydrates to generate energy and biosynthetic intermediates.

Of note, cancer cells commonly exhibit the Warburg effect, specifically by increasing glucose utilization through glycolysis, with a concomitant decrease in oxidative phosphorylation [12,13]. To maintain their redox poise, cancer cells shunt pyruvate through lactate dehydrogenase (LDH). In other words, the fermentative metabolism of *S. mitis/oralis* is very similar to that of cancer cells. OXA, a pyruvate analog that is a competitive inhibitor of LDH, is being investigated as an anti-neoplastic drug to inhibit the Warburg effect [13]. Based on our recent delineation of *S. mitis/oralis* metabolism and the changes associated with DAP resistance [9], we hypothesized that OXA would likely alter growth and susceptibility to DAP in this VGS subgroup [9].

## 2. Materials and Methods

### 2.1. Bacterial Strains

The well-characterized DAP-S *S. mitis/oralis* strain, 351, and its in vitro passage–derived isogenic DAP-R variant, 351-D10, were used in this study [14]. The parental strain 351 was isolated from the bloodstream of a patient with IE; it is resistant to penicillin (MIC = 8 μg/mL) and susceptible to ceftriaxone and ceftaroline (MIC = 8 and 0.5 μg/mL, respectively). Strain 351-D10 was generated following 10 days of in vitro passage with DAP (16); its penicillin, ceftriaxone, and ceftaroline MICs were 4 (resistant), 4 (susceptible), and 2 (susceptible) μg/mL, respectively.

### 2.2. Cultivation Conditions

The cultivation conditions for these strains have been described [15,16,17,18]. Briefly, *S. mitis/oralis* strains were grown in Bacto Brain Heart Infusion (BHI) medium, BHI + 2 µg/mL D-Glucose (BHI + glucose), or on blood agar plates (VWR Scientific Radnor, PA, USA) in selected studies. Bacterial pre-cultures were inoculated (1:10) from overnight cultures into 50 mL of BHI in 50 mL conical tubes and incubated at 37 °C, without shaking, for 4 h. These exponential-growth-phase pre-cultures were collected by centrifugation at 4000 rpm and 22 °C and suspended in 1 mL of culture medium. Primary cultures were inoculated into 50 mL (BHI + glucose) with or without OXA in 50 mL conical tubes to an absorbance at 600 nm (A_600_) of 0.16 and incubated at 37 °C. Cultures were mixed by inversion every 30 min prior to sampling. The A_600_ and pH were recorded every 30 min for 5 h. 

### 2.3. Determination of Glucose and Lactate Concentrations in Cultivation Media

Cell-free media were collected hourly by centrifugation, transferred to 1.5 mL microcentrifuge tubes, and stored at −20 °C until use. Glucose and lactate concentrations in the culture media were quantified from three biological replicates with kits purchased from R-Biopharm (Glucose Test Kit, #10716251035; and L-Lactic Acid Test Kit, #10139084035).

### 2.4. Lactate Dehydrogenase (LDH) Assay

Bacteria were cultivated as described; bacteria were harvested hourly by centrifugation and disrupted by using a Fast Prep instrument (FP120 Thermo Savant). Cell debris was removed by centrifugation. The cell-free lysate was used for the LDH activity assays (Sigma-Aldrich MAK066 Kit). LDH activity was measured from three biological replicates. Protein concentrations were determined by using the Bradford protein assay (Fisher Scientific, Waltham, MA, USA). 

### 2.5. Minimum Inhibitory Concentrations (MICs)

DAP was obtained from Merck & Co., Inc. (Whitehouse Station, NJ, USA). DAP MIC testing was performed by the CLSI-recommended broth microdilution techniques, with 50 µg/mL of CaCl_2_ added to BHI broth (Difco). In addition, BHI agar supplemented with 5% lysed horse blood (Difco) was used for quantitative agar-plate colony counts. DAP MICs were also determined in parallel by standard Etest (Biomerieux) on Mueller–Hinton agar (MHA) plates (Difco Laboratories Detroit, MI, USA). For VGS, there are no formal DAP breakpoints; however, streptococcal strains with DAP MICs ≥ 2 µg/mL are considered as DAP-R [15]. The MICs of OXA against *S. mitis/oralis* strains 351 and 351-D10 were determined by microbroth dilution methods; no formal breakpoints exist for this agent. At least three independent microbroth-dilution-based MICs were performed on distinct days for all agents. The DAP MICs of *S. mitis/oralis* strains 351 and 351-D10 were 0.5 and >256 µg/mL, respectively, as assessed by both microbroth dilution and ETEST methodologies (Table 1). Both 351 and 351-D10 strains had identical high MICs to OXA (Table 1). 

### 2.6. Time–Kill Curves

In vitro time–kill curves were performed to determine the bactericidal impacts of DAP or OXA alone vs. combined DAP + OXA. DAP or OXA were utilized at or below their respective in vitro MICs (either 0.5 or 1×). As *S. mitis/oralis* strains are capnophilic and slow-growing, we utilized an overnight BHI culture, which was diluted to a final initial inoculum of ~1–2 × 10^5^ CFU/mL (28). At 0, 2, 4, 6, 8, and 24 h post-inoculation, aliquots were obtained and quantitatively cultured onto agar plates (28, 29). Quantitative data were calculated as the mean log_10_ CFU/mL (+/− SD) of surviving counts. A minimum of three biological replicates were independently performed on separate days. A “bactericidal effect” was defined as ≥3 log_10_ CFU/mL reduction in counts relative to the initial inoculum; a “synergistic effect” was defined as ≥2 log_10_ CFU/mL reduction in counts at the 24 h sampling, when comparing the combined DAP + OXA vs. DAP or OXA alone [15,16].

### 2.7. In Vitro Resensitization to DAP

To assess the potential of OXA to reverse resistance to DAP, strain 351-D10 was serially passaged for 10 days in the presence of OXA (2048 µg/mL). Three biological replicates were performed. DAP MICs were then performed as described on these post-passage strains.

### 2.8. Ex Vivo Simulated Endocarditis Vegetation (SEV) Model

The SEV pharmacokinetic–pharmacodynamic (PK–PD) system has the ability to provide a human-equivalent model of IE, as well as to simulate human antibiotic concentrations that precisely mimic clinically relevant therapeutic regimens [17,18,19,20]. SEVs were prepared as described [17,18,19,20] by mixing cryoprecipitate as the source of fibrin, a platelet suspension (American Red Cross), aprotinin (Sigma-Aldrich), and a suspension of each *S. mitis/oralis* strain-of-interest in separate experiments (final inoculum, ~10^9^ CFU/0.5 g of SEV). This methodology results in SEVs that contain approximately 3.8–3.9 g/dL of total protein. The resultant SEV mixture was then clotted by using bovine thrombin in siliconized micro-centrifuge tubes (Pfizer, New York City, NY, USA). Infected SEVs were removed from the tubes and suspended in the chamber model (~250 mL of media) via a sterile monofilament line. Fresh medium was continuously added (along with DAP and/or OXA), and spent media were removed from the model via a peristaltic pump (Masterflex, Cole-Parmer Instrument Company, Chicago, IL, USA) at a rate which was set to simulate the half-lives of the drugs of interest. For the SEV model, due to the dependency of DAP on calcium for antimicrobial activity and calcium loss from the media due to calcium binding to albumin, the media were supplemented to a concentration of 75 µg/mL of CaCl_2_, as previously described [17,18,19,20].

At pre-assigned times over a 48 h exposure period, SEVs were removed from the chamber model, weighed, homogenized, and quantitatively cultured to determine bacterial counts. Tryptic soy agar supplemented with 5% lysed sheep’s blood (Difco) was used for quantitative sub-culturing from the SEV models. To maximize subculture yields, all plates were incubated in an anaerobic chamber for 18–24 h at 37 °C before performing colony counts. Bacterial counts were graphed vs. time over 48 h to evaluate potential bactericidal and synergistic activities of DAP or OXA alone, as well as combination regimens, using the same metrics as delineated above.

The limit of detection for these samples was 2 log_10_ CFU/g. To determine the comparative antibacterial activities of the various regimens within SEVs, the results were plotted, (log_10_ CFU/g vs. time (h)) via Prism, GraphPad 8.0.0; San Diego, CA, USA).

### 2.9. Pharmacokinetic (PK) Studies

For the 48 h SEV treatment duration, we utilized PK modeling for human-equivalent DAP-alone dose regimens (6 mg/kg/; once-daily) vs. DAP + OXA (2 g by continuous infusion). This DAP dose-regimen is the current FDA-approved therapeutic recommendation for serious infections.

DAP was administered once daily via the chamber’s injection port. The simulated DAP regimens represented a targeted T_1/2_ of 8 h at 6 mg/kg/d, with a targeted peak concentration of ~94 µg/mL. OXA was administered by continuous infusion of 2.048 µg/mL based on the in vitro–defined MICs. Published standard human-achievable and/or therapeutic blood-level dosage regimens of OXA were used to guide our dosing strategies [17,18,19,20]. All models were performed in duplicate to ensure reproducibility. 

Sampling for PK assays was performed by removing 1 mL of chamber fluid media (through the injection port) from each of the two parallel SEV models at 0, 2, 4, 6, 8, 32, or 48 h, in duplicate, as previously described [20]. DAP PK evaluations were confirmed by a validated high-performance liquid chromatography (HPLC) assay that conforms to the guidelines set forth by the College of American Pathologists [17,18,19,20]. This technique demonstrated intra-day coefficients of variation of between 0.56% and 7.14% for all DAP standards. The DAP peak, AUC_0–24h_ and half-life metrics were determined by the trapezoidal method, utilizing standard PK modeling software (PK analyst version 1.1; MicroMath Scientific Software, Salt Lake City, UT, USA). 

In the SEV model, simulated once-daily DAP dosing at 6 mg/kg achieved a C_max_ of 94.23 ± 3.21 µg/mL, AUC_0–24h of_ 1091.03 ± 78.01 (µg/mL), t_1/2_ of 8.17 ± 0.23 h (targeted C_max_ and t_1/2_ = 93.9 µg/mL and 8 h, respectively, for DAP) [21,22]. 

### 2.10. Pharmacodynamic (PD) Analyses

For PD evaluations, two SEVs were aseptically removed from each model (total = 4 samples) at 0, 4, 8, 32, and 48 h. Pilot studies confirmed these time points as providing the most consistent and differential microbiologic readouts, using this *S. mitis/oralis* strain pair. After weighing, each SEV was homogenized with trypsin, serially diluted with cold normal saline (to minimize antibiotic carryover), and then plated for quantitative cultures. The same metrics of “bactericidal” and “synergistic” outcomes as defined above for in vitro assays were employed for SEVs. Graphs were prepared by using Prism (9.1.0) software.

### 2.11. Emergence of DAP-R in the SEV Model

The potential emergence of DAP resistance over the 48 h exposures to DAP alone vs. DAP–OXA combinations within the SEV model was evaluated. Serial SEV isolates were parallel-plated on antibiotic-free and DAP-containing plates (3 × the baseline MIC of the parental DAP-S strain). The DAP MICs of colonies detected on DAP-containing plates were then confirmed by using the above microbroth dilution technique according to CLSI guidelines.

#### Statistical Analysis

Means and standard deviations (SDs) were calculated for all variables. Differences between strains for in vitro killing and metabolic biochemical assays were analyzed with the two-tailed Student’s *t* test. One-way ANOVA with Tukey’s post hoc test (Prism 9.1.0) software was applied to compare PD outcomes in the SEV model regarding the impact of DAP and DAP + OXA upon log_10_ CFU/g comparative results. The *p*-values ≤ 0.05 were considered to be “significant”.

## 3. Results and Discussion

### 3.1. OXA Inhibits S. mitis/oralis Growth

**Figure 1:**
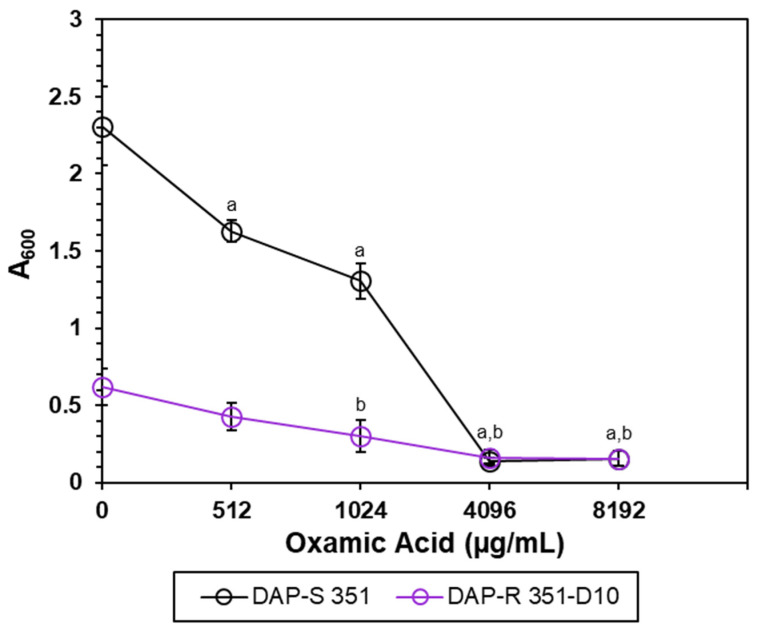
OXA decreases the growth yields of *S. mitis/oralis* strains 351 and 351-D10. Bacteria were cultivated with increasing concentrations of OXA at 5 h post-inoculation, and the growth yields were determined spectrophotometrically (A_600_). Data represent the mean (+/− SD) of three independent experiments. Strains 351 and 351-D10 controls vs. OXA were determined by using Student’s *t*-test; ^a^ *p* < 0.05 351 Control vs. OXA; ^b^ *p* < 0.05 351-D10 Control vs. OXA.

**Figure 2:**
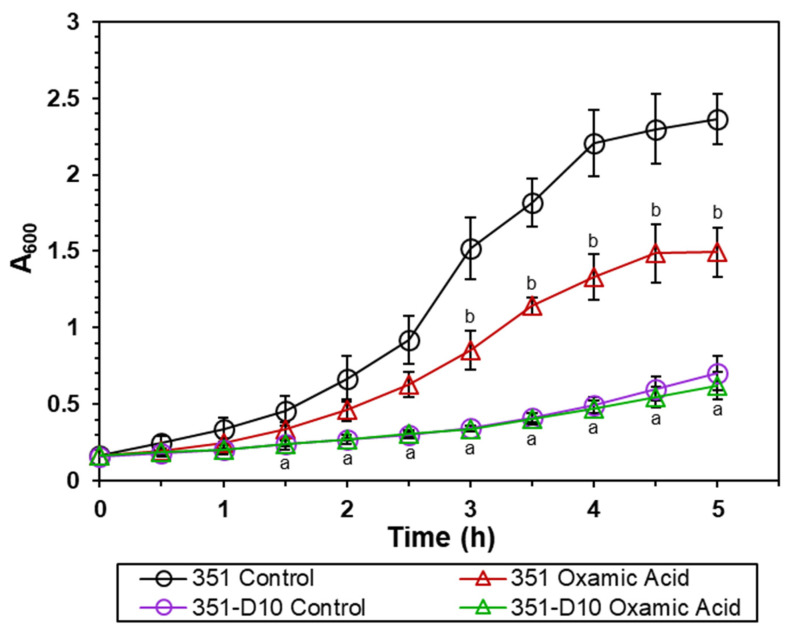
OXA decreases the growth rate of the *S. mitis/oralis* strain. Growth (A_600_) patterns were assessed hourly. Data represent the mean (+/− SD) of three independent experiments. Statistical differences for strain 351 control relative to strain 351-D10, 351 +/− OXA, and 351-D10 +/− OXA were determined by Student’s *t*-test; ^a^ *p* < 0.05 351 control vs. 351-D10; ^b^ *p* < 0.05 351 control vs. 351 OXA.

Cultivation of *S. mitis/oralis* strains 351 and 351-D10 with increasing concentrations of OXA dramatically decreased the 5 h growth yield of strain 351, while further reducing that of strain 351-D10 (Figure 1). One possibility for this outcome was that OXA affects the growth rate, which would decrease the number of bacterial doublings over the 5 h cultivation period, leading to lower growth yields. To test this possibility, the growth rates of strains 351 and 351-D10 were assessed in the presence of OXA (Figure 2). OXA decreased the growth rate of DAP-S strain 351 relative to that of an untreated culture. In contrast, OXA (4096 µg/mL) did not alter the growth rate of strain 351-D10, which is already slower than the parental strain 351.

### 3.2. OXA Decreases Glucose/Pyruvate Catabolism

**Figure 3:**
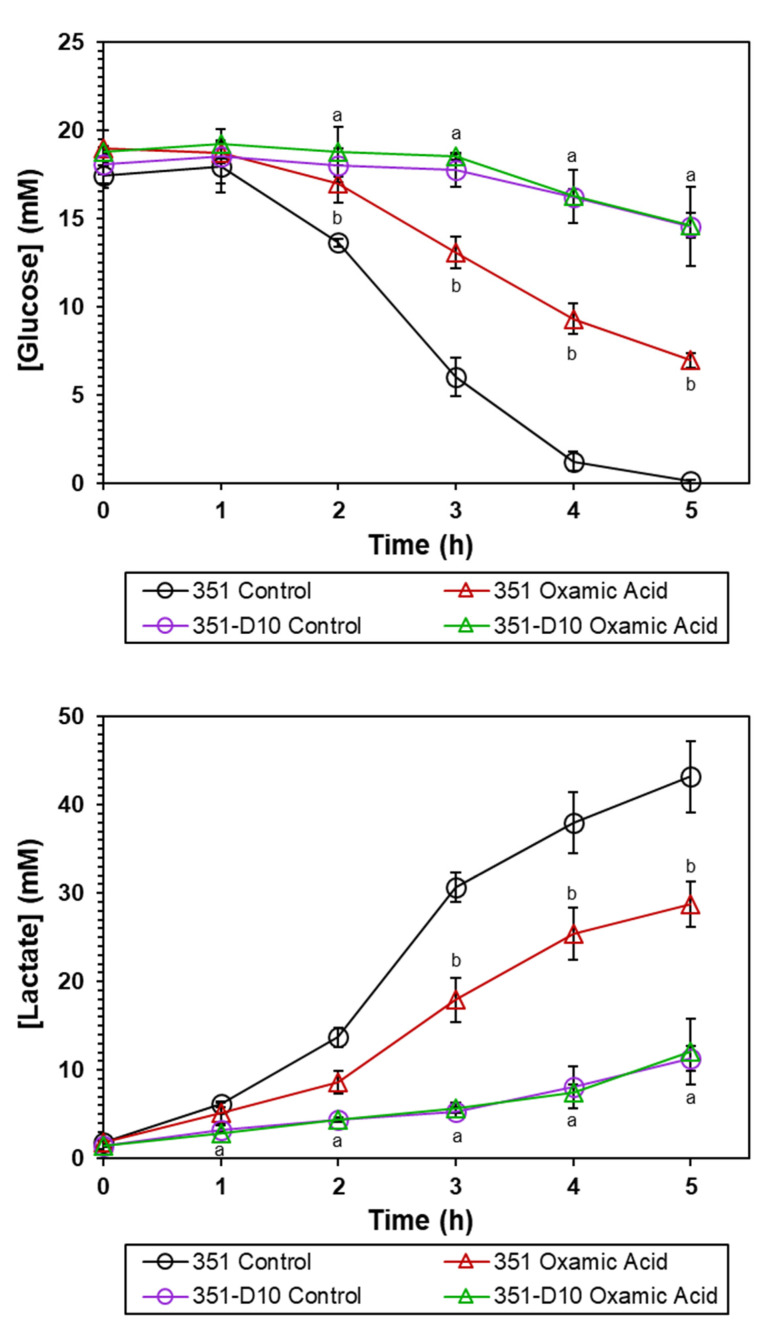
OXA alters glucose depletion and lactate accumulation of *S. mitis/oralis* strains 351 and 351-D10. Glucose depletion and lactate accumulation were assessed hourly. Data represent the mean (+/− SD) of three independent experiments. Statistical differences for strain 351 control relative to strain 351-D10, 351 +/− OXA, and 351-D10 +/− OXA were determined by Student’s *t*-test; ^a^ *p* < 0.05 351 control vs. 351-D10; ^b^ *p* < 0.05 351 control vs. 351 OXA.

The more likely explanation for the effect of OXA on *S. mitis/oralis* growth (Figure 2) is that it decreases the ability of *S. mitis/oralis* to catabolize glucose. This would occur because OXA is an inhibitor of the enzyme LDH. Inhibiting LDH prevents the oxidation of NADH to NAD^+^, which, in turn, decreases the availability of reduced dinucleotides that are necessary for glycolysis (see Supplementary Figure S1). Thus, glucose utilization would slow, causing the growth rates and growth yields to decrease. In this scenario, the effect of OXA on strain 351-D10 would be greatly reduced because LDH activity is already impaired [9]. To assess the effect of OXA on glycolysis, both glucose consumption and lactate accumulation were followed over time during growth of *S. mitis/oralis* strains 351 and 351-D10 (Figure 3). As expected, OXA slowed the utilization of glucose by the DAP-S strain 351 (but not by the DAP-R strain 351-D10). This OXA-induced decrease in glucose utilization translated into decreased lactate accumulation in the culture medium of strain 351 (Figure 3). These data strongly suggest that OXA is acting through LDH to alter carbon flow through glycolysis.

### 3.3. OXA Decreases LDH Activity

**Figure 4:**
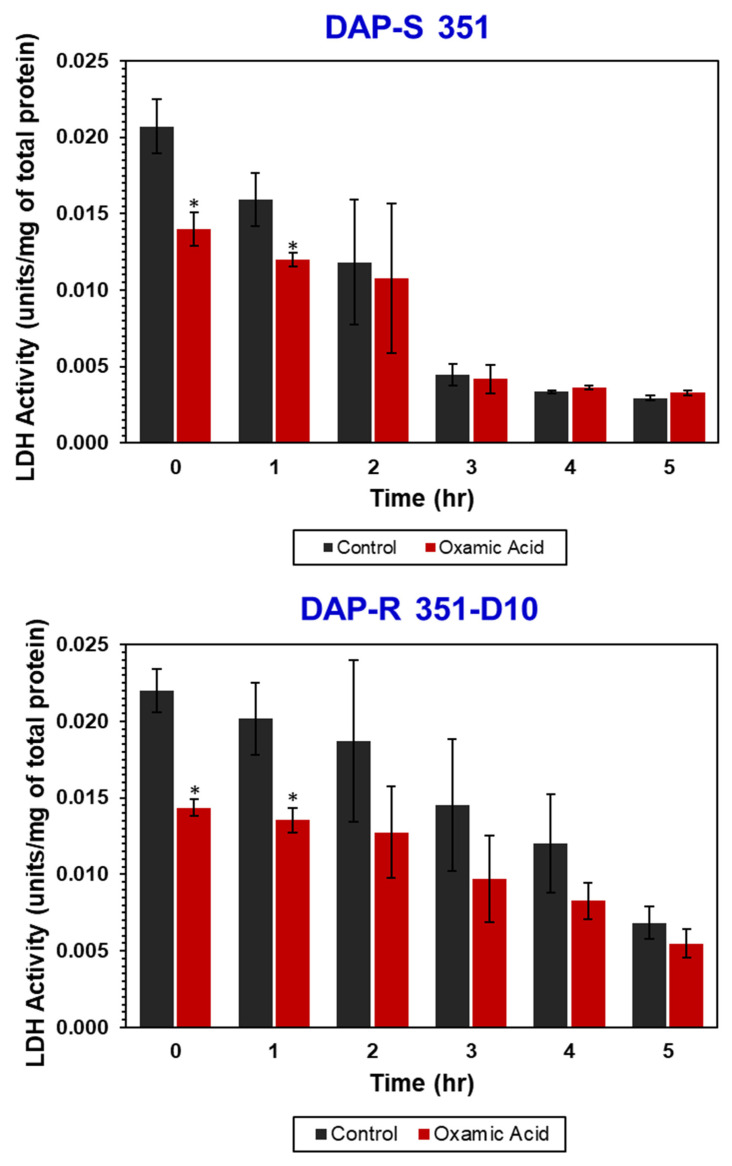
Temporal LDH activity of *S. mitis/oralis* strains 351 and 351-D10. LDH activity was assayed from cell-free lysates from cultures cultivated in BHI and harvested at multiple time points. The data represent the mean (+/− SD) of three independent biological replicates. Statistical significance (*) was assessed by using Student’s *t*-test (*p* ≤ 0.05); * *p* < 0.05 351 or 351-D10 controls vs. OXA.

To directly assess the effect of OXA on LDH activity, *S. mitis/oralis* strains 351 and 351-D10 were cultivated for 0–5 h, with or without OXA, and then the LDH activity was assessed (Figure 4). Consistent with the growth profiles, as well as the glucose-depletion and lactate-generation data above (Figure 3), OXA decreased LDH activity relative to untreated cultures in both strains (Figure 4). Because OXA is a competitive inhibitor that reversibly binds to enzymes, a large decrease in enzymatic activity was not anticipated because the enzyme assay supplies the substrate in abundance. Surprisingly, the baseline LDH activity in strain 351-D10 was equivalent to that of strain 351; this suggested that decreased glucose consumption and lactate accumulation were growth-related and not actually due to decreased LDH activity. Taken together, these data suggest that OXA decreases *S. mitis/oralis* LDH, which then decreases glucose flow through glycolysis and NADH oxidation. It should also be noted that the transcription and translation of LDH and glycolytic enzymes are tightly linked with the glucose concentration in the medium, which would alter LDH activity over time.

### 3.4. OXA Is Bactericidal against Both DAP-S and DAP-R Strains

**Figure 5:**
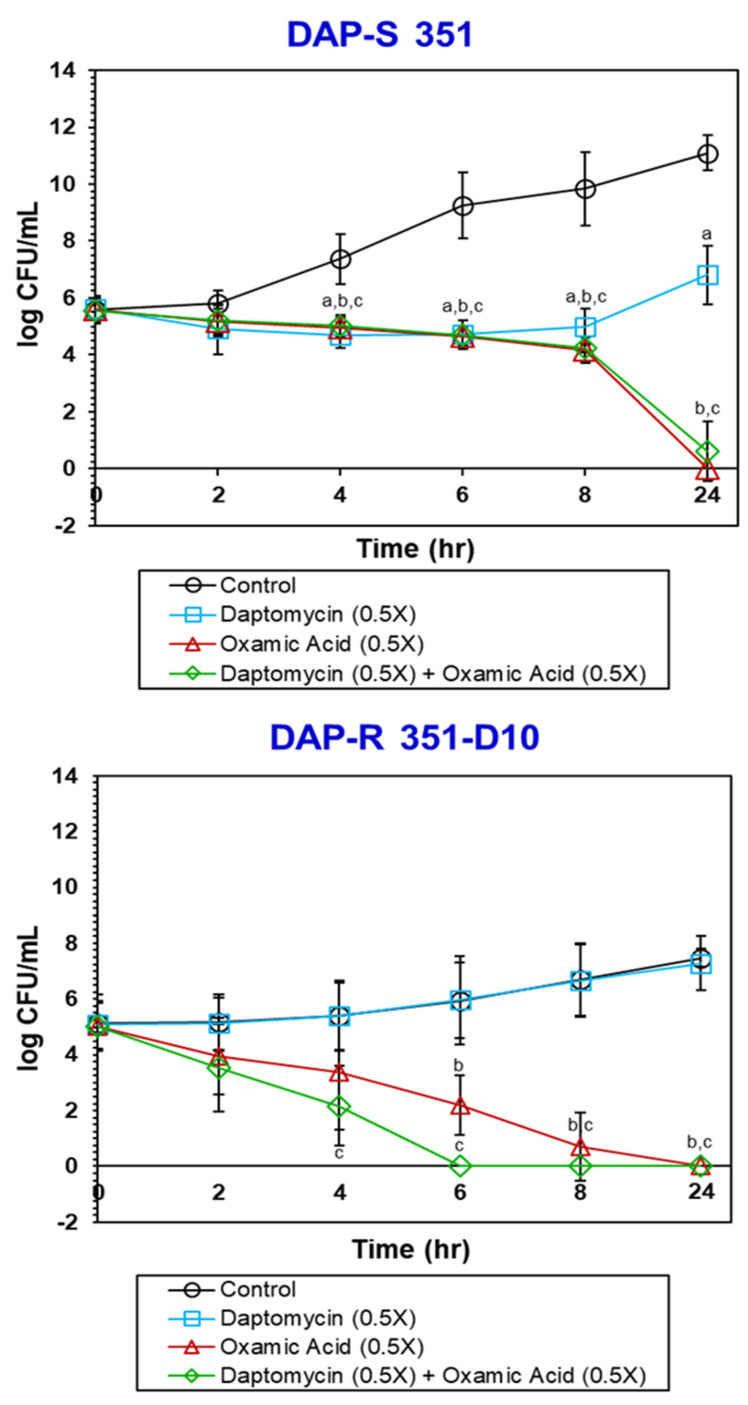
OXA is bactericidal against *S. mitis/oralis* strains 351 and 351-D10 in the in vitro time–kill experiments. The data represent the mean (+/− SD) from three biological replicates. Statistical significance (a, b, c) was assessed by using Student’s *t*-test (*p* ≤ 0.05); ^a^ *p* < 0.05 Control vs. DAP (0.5×); ^b^ *p* < 0.05 Control vs. OXA (0.5×); ^c^ *p* < 0.05 Control vs. DAP + OXA (0.5×).

**Figure 6:**
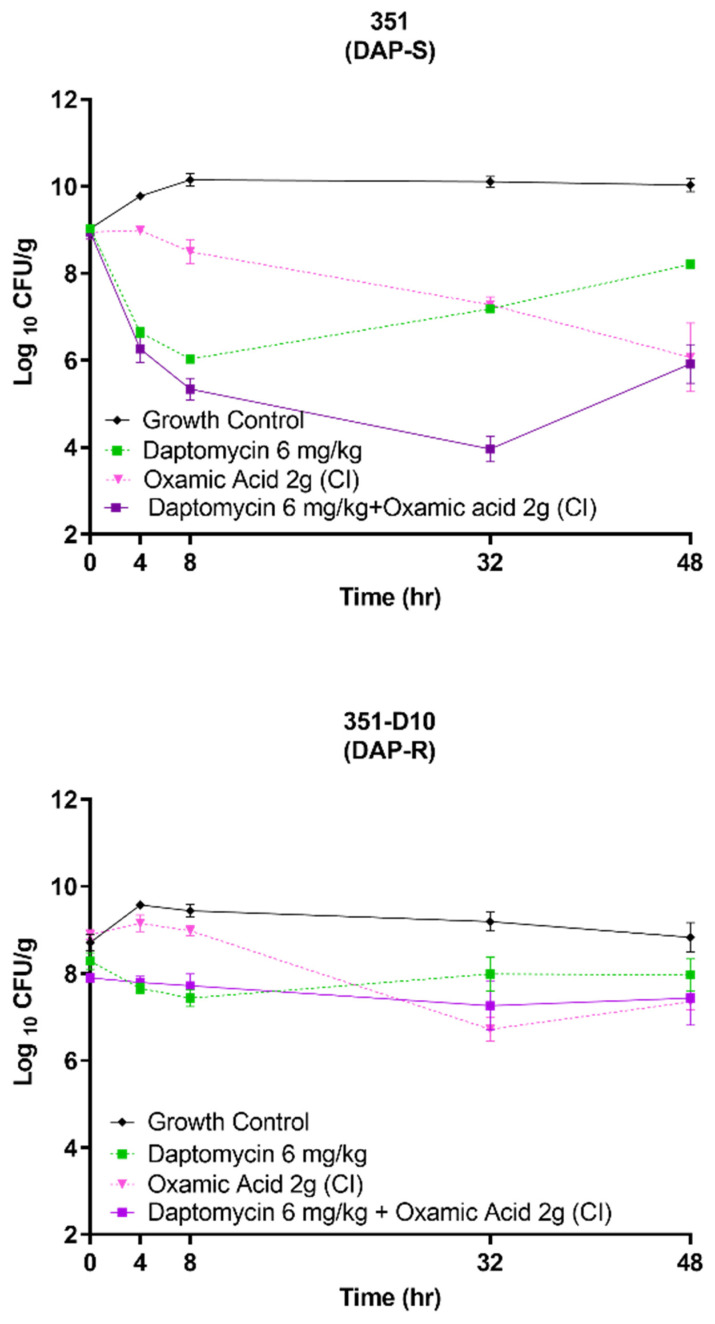
Comparison of DAP + OXA combinations vs. DAP-alone in SEVs ex vivo. The data represent the means (+/− SD) from two independent runs with four biological replicates. CI = continuous infusion.

As noted above, OXA is growth inhibitory against *S. mitis/oralis* strains 351 and 351-D10 (Figure 1 and Figure 2). This could be due to its being either bacteriostatic or bactericidal. To determine which of these possibilities was more likely, in vitro and ex vivo kill curves were determined for the two strains (Figure 5 and Figure 6). In vitro, OXA alone or combined with DAP was bactericidal (>3 log_10_ CFU/mL reductions in counts) against both strains (Figure 5). Ex vivo, for the DAP-S 351 strain, the combination of DAP + OXA was rapidly bactericidal and synergistic over the first 32 h of exposure (Figure 6). After 48 h of exposure, both the OXA alone and the DAP–OXA combination remained bactericidal. In contrast, for the DAP-R 351-D10 strain, both OXA and DAP–OXA in combination exerted only a bacteriostatic effect ex vivo. This latter result is likely due to the slower growth of strain 351-D10 relative to strain 351 (Figure 2).

### 3.5. OXA Prevents the Emergence of the DAP-Resistance Phenotype

We have shown that altering the metabolic capabilities of *S. aureus* with targeted metabolic inhibitors can prevent the emergence of DAP resistance and/or reverse preexisting DAP resistance [10]. To determine if OXA could similarly reverse DAP resistance in *S. mitis/oralis* strain 351-D10, strain 351-D10 was cultivated by serial passage with OXA for 10 d, and the DAP MICs were determined. The MICs of strain 351-D10 colonies did not change over the 10 d OXA exposure period (*data not shown*). Similar to the in vitro serial passage of strain 351-D10, the combination of DAP and OXA in the SEV model did not reverse DAP resistance (*data not shown*).

To assess the ability of OXA to prevent the emergence of DAP-R, strain 351 was serially passaged with DAP (20 µg/mL) or DAP plus OXA, and then DAP MICs were determined. Following serial passage with DAP +/− OXA, all colonies examined had acquired DAP-R (data not shown). In contrast, in the SEV model, cultivation of *S. mitis/oralis* strain 351 with DAP + OXA over 48 h prevented the emergence of DAP resistance (DAP MIC = 0.5 µg/mL) seen with DAP-alone exposures (DAP MIC ≥ 64 µg/mL). These data suggest that in vitro resensitization to DAP and preventing the emergence of DAP resistance are likely to represent two discrete mechanistic events involving separate metabolic pathways; this concept is in agreement with our previous observations related to the evolution of DAP resistance in *S. aureus* strains (10).

## 4. Conclusions

*S. mitis/oralis* has incomplete central metabolism (i.e., it lacks the TCA cycle [9]). This causes *S. mitis/oralis* to rely on glycolysis for energy and a limited set of biosynthetic intermediates, as well as on LDH for redox balance.

This metabolic scenario is similar to that of cancer cells, where the Warburg effect is common [12]. Targeting cancer cells displaying the Warburg effect with LDH inhibitor has driven interest in OXA and its derivatives for use as anti-neoplastic drugs [23]. Not only are cancer cells susceptible to OXA, but, based on our current investigations, fermentative bacteria, such as *S. mitis/oralis* are, as well (Figure 4). Of particular importance, OXA not only synergistically kills DAP-S *S. mitis/oralis* strains in combination with DAP, but it also prevents the emergence of DAP resistance in a host-mimicking microenvironment that simulates a human endovascular infection. This is of note, given the propensity of DAP-S *S. mitis/oralis* strains to evolve rapid, high-level, and durable DAP resistance when exposed to DAP [1].

These data raised the notion that OXA-like compounds could make effective small molecule inhibitors of *S. mitis/oralis* and other fermentative bacteria. Of note, the potential for OXA as an adjunctive treatment is being actively explored in *Mycobacterium tuberculosis*, where OXA derivatives are being used to target protein tyrosine phosphatases [24]. As additional OXA derivatives are developed to treat cancer, it is likely that some of these compounds may also be effective at killing fermentative bacterial pathogens, such as *S. mitis/oralis*.

It should be noted that our study had some important limitations: (i) only a single strain pair of DAP-S/DAP-R *S. mitis/oralis* was utilized. A larger strain cohort will be required to assess the impacts of the OXA more fully and statistically on DAP-resistance phenotypes; and (ii) our differential in vitro vs. ex vivo findings, which show distinct outcomes in the host-mimicking ex vivo microenvironment, need in vivo validation in endovascular infection models (e.g., experimental endocarditis). These studies are still in progress.

## Figures and Tables

**Table 1:** MICs of 351 and 351 D10 *S. mitis/oralis* against DAP and OXA.

DAP MIC (µg/mL)		
Strains	DAP	OXA
DAP-S 351	0.5	2048
DAP-R 351D10	>256	2048

## Data Availability

Not applicable.

## Supplementary Materials

The following supporting information can be downloaded at: https://www.mdpi.com/article/10.3390/antibiotics11101409/s1, Figure S1: Oxamic acid and its site of action.
