# Supplementary figures and images for: Activity of the Lactate Dehydrogenase Inhibitor Oxamic Acid against the Fermentative Bacterium Streptococcus mitis/oralis: Bactericidal Effects and Prevention of Daptomycin Resistance In Vitro and in an Ex Vivo Model
Source: Antibiotics (Basel). 2022 Oct 13;11(10):1409. doi: 10.3390/antibiotics11101409 (PMC9598551; doi:10.3390/antibiotics11101409)

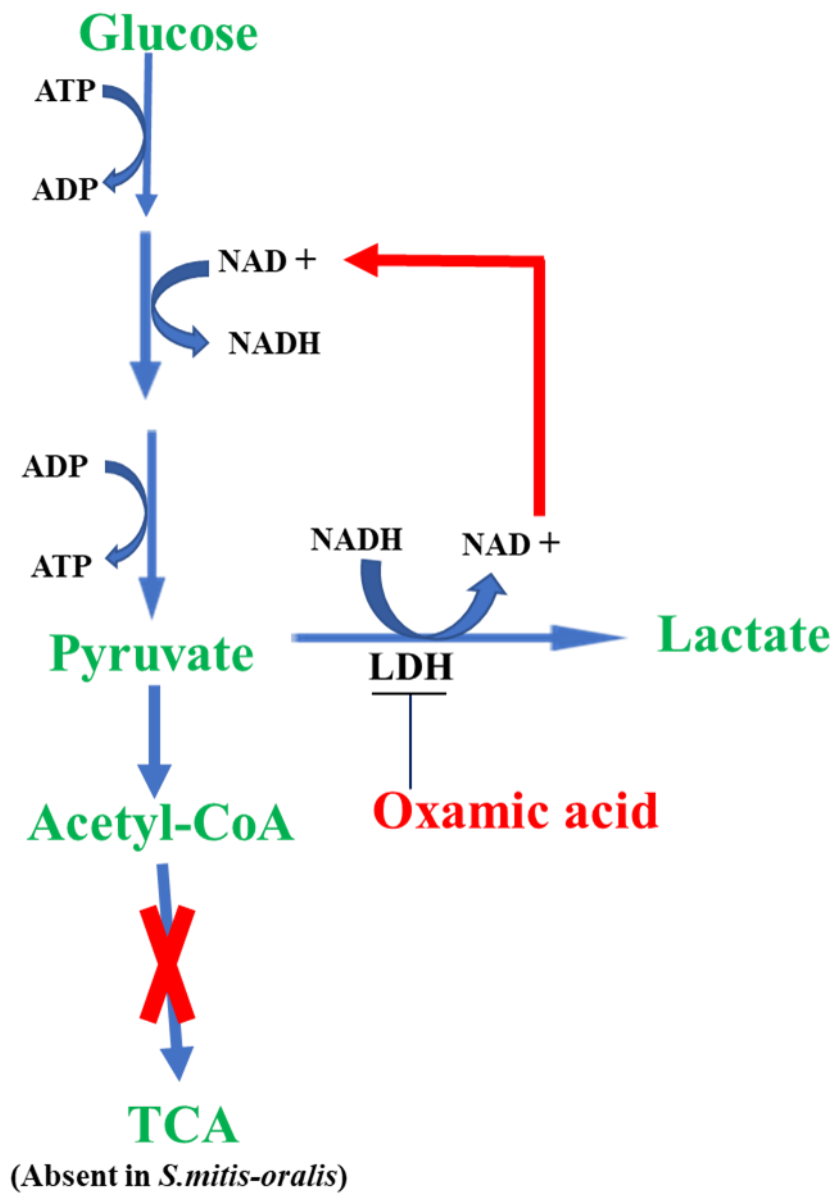

Supplementary Figure S1. Oxamic acid and its site of action

Supplement: Supplementary file 1 [file antibiotics-11-01409-s001.zip › antibiotics-1896194-supplementary.pdf]
